# Rapid Audiovisual Integration Guides Predictive Actions

**DOI:** 10.1523/ENEURO.0134-23.2023

**Published:** 2023-08-25

**Authors:** Philipp Kreyenmeier, Anna Schroeger, Rouwen Cañal-Bruland, Markus Raab, Miriam Spering

**Affiliations:** 1 Department of Ophthalmology & Visual Sciences, University of British Columbia, Vancouver, British Colombia V5Z 3N9, Canada; 2 Graduate Program in Neuroscience, University of British Columbia, Vancouver, British Colombia V6T 1Z2, Canada; 3 Department of Psychology, Justus Liebig University Giessen, 35390 Giessen, Germany; 4 Department for the Psychology of Human Movement and Sport, Friedrich Schiller University Jena, 07743 Jena, Germany; 5 Department of Performance Psychology, German Sport University Cologne, 50933 Cologne, Germany; 6 School of Applied Sciences, London South Bank University, London SE1 0AA, United Kingdom; 7 Djavad Mowafaghian Centre for Brain Health, University of British Columbia, Vancouver, British Colombia V6T 1Z3, Canada; 8 Institute for Computing, Information, and Cognitive Systems, University of British Columbia, Vancouver, British Colombia V6T 1Z4, Canada

**Keywords:** eye movements, interception, multisensory integration, perception-action, prediction

## Abstract

Natural movements, such as catching a ball or capturing prey, typically involve multiple senses. Yet, laboratory studies on human movements commonly focus solely on vision and ignore sound. Here, we ask how visual and auditory signals are integrated to guide interceptive movements. Human observers tracked the brief launch of a simulated baseball, randomly paired with batting sounds of varying intensities, and made a quick pointing movement at the ball. Movement end points revealed systematic overestimation of target speed when the ball launch was paired with a loud versus a quiet sound, although sound was never informative. This effect was modulated by the availability of visual information; sounds biased interception when the visual presentation duration of the ball was short. Amplitude of the first catch-up saccade, occurring ∼125 ms after target launch, revealed early integration of audiovisual information for trajectory estimation. This sound-induced bias was reversed during later predictive saccades when more visual information was available. Our findings suggest that auditory and visual signals are integrated to guide interception and that this integration process must occur early at a neural site that receives auditory and visual signals within an ultrashort time span.

## Significance Statement

Almost all everyday actions, from catching a ball to driving a car, rely heavily on vision. Although moving objects in our natural visual environment also make sounds, the influence of auditory signals on motor control is commonly ignored. This study investigates the effect of sound on vision-guided interception. We show that sound systematically biases interception movements, indicating that observers associate louder sounds with faster target speeds. Measuring eye movements during interception revealed that vision and sound are integrated rapidly and early in the sensory processing hierarchy. Training and rehabilitation approaches in sports and medicine could harness the finding that interceptive hand movements are driven by multisensory signals and not just vision alone.

## Introduction

When intercepting a rapidly moving object with our hands—swatting a fly or catching a ball—we rely heavily on vision. Humans and other animals direct their eyes at moving objects of interest to sample critical visual information, such as the position of the object, speed, and acceleration (Kreyenmeier et al., 2022; Brenner et al., 2023), and to increase performance accuracy (Spering et al., 2011; Diaz et al., 2013; Borghuis and Leonardo, 2015; Michaiel et al., 2020; Fooken et al., 2021). However, other sensory modalities also supply information that might be used to guide behavior in interception tasks. Indeed, in goalball—an interceptive sport for visually impaired athletes—players rely solely on auditory information to locate and intercept a ball (https://goalball.sport/wp-content/uploads/2022/04/IBSA-Goalball-Rules-and-Regulations-2022-2024-v1.1-4-Feb-22.docx-Summary-Change-document.pdf). Our study addresses the question of whether and under which conditions vision-guided interceptive actions rely on sound information in normally sighted observers.

In our natural environment, object motion is almost always accompanied by sound, which can alter visual motion judgements (Sekuler et al., 1997; Soto-Faraco et al., 2003; Senna et al., 2015; Carlile and Leung, 2016; Meyerhoff et al., 2022; Wessels et al., 2022). For instance, hitting a ball with a bat or racket creates an impact sound, and its volume provides information about hit intensity and launch speed. Accordingly, impact sounds can bias perceived ball-bounce locations and perceptual ball speed judgements, suggesting that observers use auditory information to predict ball trajectories (Cañal-Bruland et al., 2018; 2022). When integrating information from different modalities, Bayesian models of multisensory integration predict that sensory signals are combined depending on the uncertainty of the different sensory signals (Ernst and Banks, 2002; Alais and Burr, 2004; Körding et al., 2007; Angelaki et al., 2009). Following this framework, auditory signals may bias visual perception most in tasks with high visual uncertainty such as when viewing conditions are poor (e.g., visual blurring of the target; Schroeger et al., 2021) or visual information is sparse (e.g., short visual presentation durations; Spering et al., 2011).

**Figure 1.**
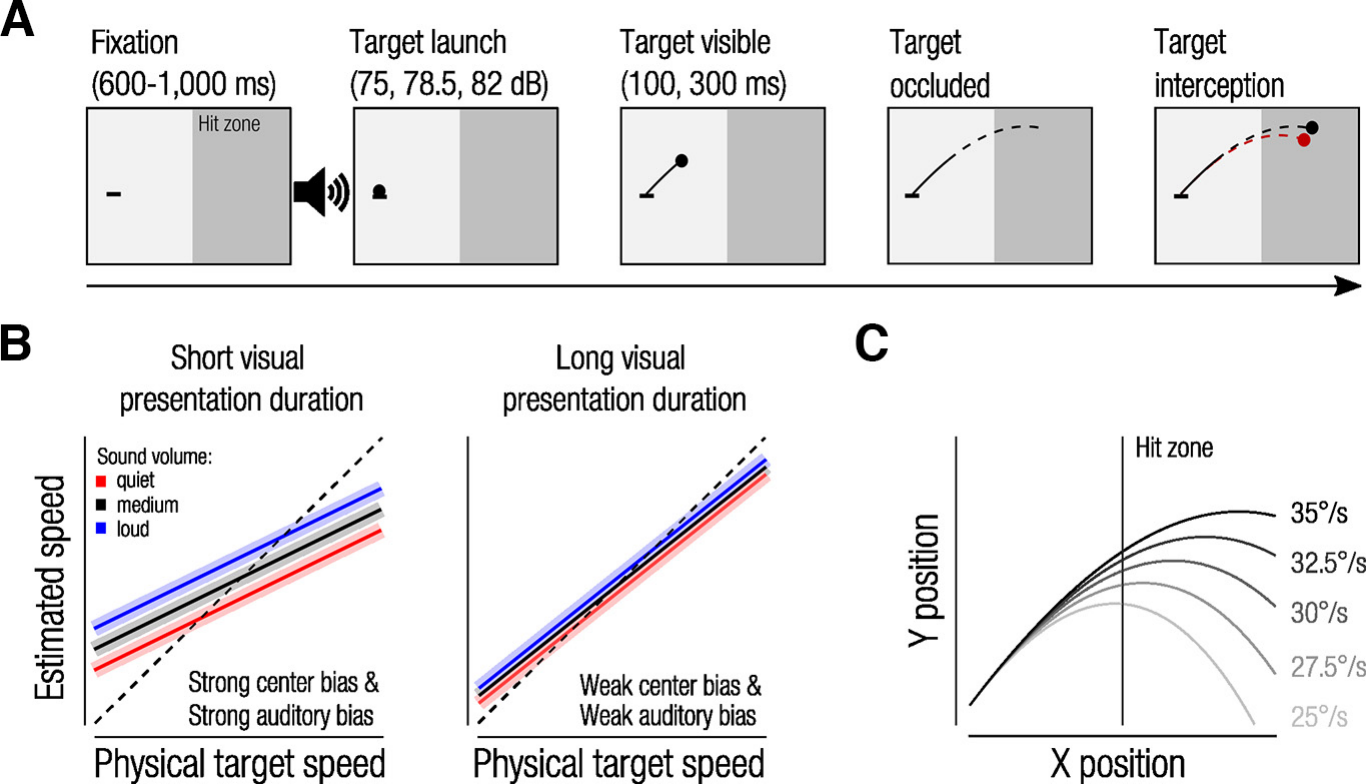
***A***, Timeline of a single trial. Black lines represent the visible (solid lines) and invisible (dashed lines) parts of the target trajectory. Observers received visual feedback of their finger position (right, red dot) and target position at time of interception (black dot). Red dashed line illustrates the trajectory that best fit the interception position. ***B***, Illustration of hypotheses. Dashed diagonal indicates veridical speed judgments. For short visual presentation durations (high visual uncertainty), we expect a strong regression in estimated speed toward the mean physical target speed (center bias). In addition, we expect that sound volume induces a systematic bias in observers’ speed estimates (slower for quiet sounds, faster for loud sounds). Conversely, for long visual presentation durations, we expect less regression toward the mean and only a weak sound-induced bias, indicating that observers relied almost entirely on visual information to estimate target trajectories. ***C***, The five presented ball trajectories defined by different initial launch speeds (gray lines). Vertical line illustrates the border of the hit zone.

Our study probes this interaction between visual uncertainty and auditory cues in a real-world-inspired, fast-paced movement interception task during which observers track the brief launch of a simulated baseball moving across the screen and intercept it at a predicted location with a quick pointing movement (Fig. 1*A*). We manipulated the sound volume of the simulated ball launch and visual uncertainty by varying the visual presentation duration of the ball. At the shortest visual presentation duration, the ball was only visible for 100 ms, a duration that pushes the perceptual system to the limits as it is close to the minimal delay of motion detectors (van de Grind et al., 1986). In this challenging task, we measured observers’ eye and hand movements toward the ball as indicators of observers’ abilities to estimate ball speed and predict the ball trajectory. We hypothesized, first, that auditory cues would systematically bias ball speed estimation. Specifically, we expected that observers would overestimate speed when the ball launch was accompanied by a loud batting sound (indicating a harder hit and higher launch speed) compared with a quiet batting sound (indicating a softer hit and lower launch speed). Second, we expected that the influence of the auditory cue would scale with visual certainty, implying that observers rely more on the auditory cue when visual presentation durations are short, in line with the assumption that auditory and visual cues are combined by weighing them according to their uncertainty (Fig. 1*B*). Further, measuring continuous eye movements during this track-intercept task allows us to investigate the time point at which auditory information interacted with visual target speed information and biased observers’ estimates of the target trajectory.

## Materials and Methods

### Participants

We show data from 16 healthy adults (25.5 ± 4.7 years; 11 females, 2 authors). This sample size was determined using an a priori power analysis in G*Power (Faul et al., 2007; power *=* 0.80; alpha *=* 0.05) with an effect size of η*_p_*^2^ = 0.34 (main effect of sound volume on estimated speed) derived from pilot data. All observers had normal or corrected-to-normal visual acuity. Study protocols were approved by the University of British Columbia Behavioral Research Ethics Board. Observers were compensated at a rate of $10 CAD per hour. 

### Apparatus

The experimental setup combined a high-resolution stimulus display with eye and hand tracking. Display and data collection were controlled by a PC (NVIDIA GeForce GTX 1060 graphics card) using MATLAB (version 9.10.0, MathWorks) and the Psychophysics and Eyelink toolboxes (version 3.0.18; Cornelissen et al., 2002; Kleiner et al., 2007). Stimuli were back projected onto a 41.8 × 33.4 cm translucent screen with a PROPixx video projector at a resolution of 1280 × 1024 pixels (120 Hz; VPixx Technologies). Two speakers (S-0264A, Logitech), located 40 cm to the left and right of the screen center, displayed the sound. Observers viewed stimuli binocularly at a distance of 44 cm while their right eye was recorded with an Eyelink 1000 Tower Mount eye tracker (1 kHz; SR Research). The 3D position of each observer’s right index finger was recorded with a 3D Guidance trakSTAR (120 Hz; Ascension Technology).

### Stimuli, experimental procedure, and design

In each trial, we displayed a small black disk that moved along a parabola, simulating the trajectory of a batted baseball affected by gravity, Magnus effect because of the spin of the ball, and aerodynamic drag force (Fooken et al., 2016; Kreyenmeier et al., 2017). The ball was launched at a constant angle of 35° at one of five launch speeds, resulting in five unique trajectories (Fig. 1*C*). All other parameters (e.g., ball mass) to simulate flyball trajectories were the same as in Fooken et al. (2016). The screen was separated into two zones by varying background luminance; the darker right side served as the hit zone in which observers were asked to intercept the ball (Fig. 1*A*). The sound of a baseball hitting a wooden bat was retrieved from a free online sound library (https://freesound.org/people/SocializedArtist45/sounds/266595/; 44.1 kHz) and played at one of three sound volumes (A-weighted sound pressure levels of 75, 78.5, or 82 dBA) for ∼50 ms, coinciding with the time of the ball launch.

Each trial began with a random-duration fixation on a line segment that marked the ball-launch position (Fig. 1*A*). After fixation, the ball was launched, paired with a batting sound at one of the three sound intensities (randomly assigned), and moved for either 100 or 300 ms before disappearing from view (Fig. 1*A*, solid black line segment). Observers were instructed to manually intercept the ball anywhere along its extrapolated trajectory (Fig. 1*A*, dashed black line segment) within the hit zone. On interception, a red dot, indicating the interception location of the finger, and a black dot, showing the actual ball position at interception, provided feedback for the observer.

Observers performed nine practice trials (six of these with the entire target trajectory visible) to familiarize themselves with the task. Batting sounds, visual presentation durations, and physical target speeds were pseudorandomly selected for each trial. The experiment consisted of 420 trials in total [14 repetitions for each possible combination of the conditions batting sound × visual presentation duration × physical target speed = 14 × (3 × 2 × 5) = 420], divided into 7 blocks of 60 trials each. Observers took short breaks between blocks.

### Eye and hand movement recordings and analyses

Eye and hand movement data were preprocessed off-line. Filtered eye movement traces [second-order Butterworth filtered with 15 Hz (position) and 30 Hz (velocity) cutoff frequencies] were aligned to the target start position.Saccades were detected when five consecutive frames exceeded a fixed velocity criterion of 30°/s. Saccade onsets and offsets were determined as the nearest reversal in the sign of acceleration before eye velocity exceeded the velocity threshold (saccade onset), and the nearest reversal in the sign of acceleration after eye velocity returned below threshold (saccade offset). We inspected all trials manually and excluded trials in which observers blinked or when the eye tracker lost the signal (3.2% of trials across participants).

**Figure 2.**
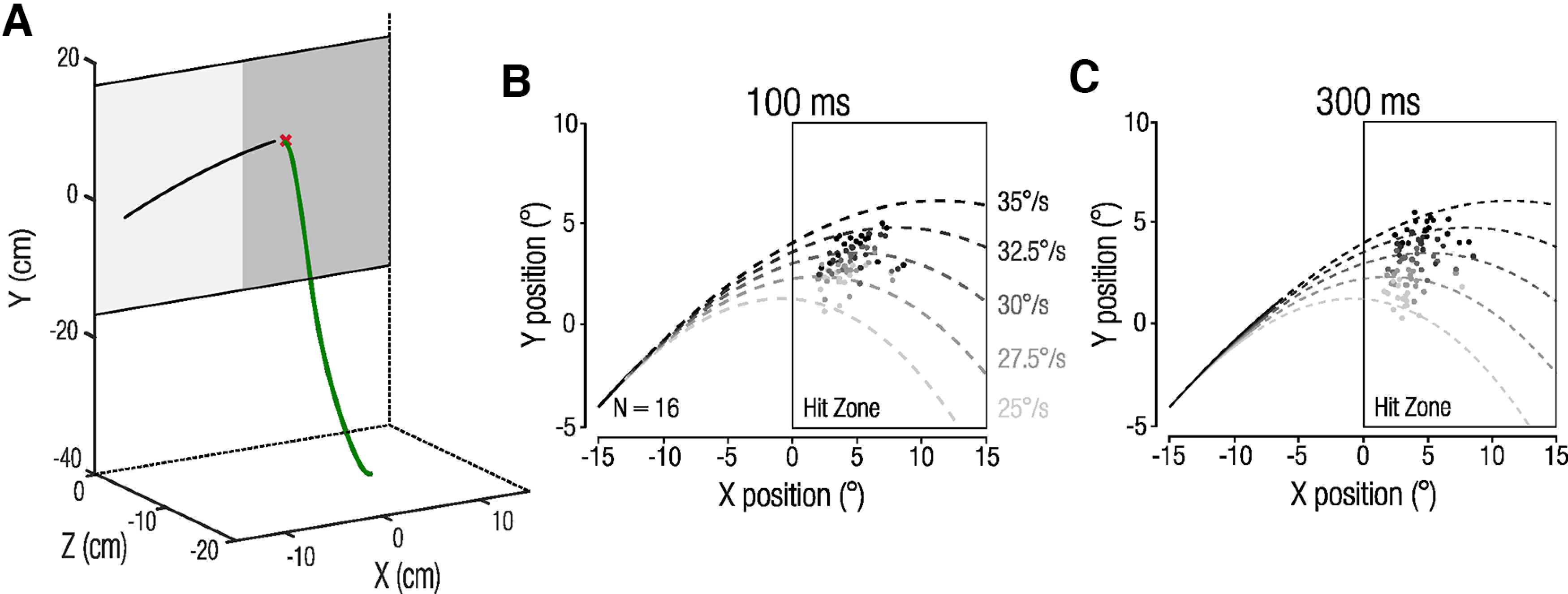
***A***, Example of a hand position trace (green). Black line represents the 2D target position, and the red cross indicates the interception position. ***B***, ***C***, Mean individual observer 2D interception positions for the 100 ms (***B***) and 300 ms (***C***) visual presentation durations. Each data point indicates one observer’s mean interception position per each of the five target speeds.

Hand position data were filtered using a second-order Butterworth filter (15 Hz cutoff) and then upsampled to 1 kHz by linear interpolation. Hand latency was computed as the first sample exceeding 5% of the peak hand velocity in that trial. Hand movement offset was detected when the finger landed within ±0.80 mm of the screen. If no interception was detected online, interception time and position were determined off-line as the maximum hand position in the *z*-dimension (depth; Fig. 2*A*).

We then used the 2D hand interception position to calculate estimated speed. For each individual trial, we determined which target trajectory best fit the observed interception position (Fig. 1*A*, red dot), as follows. We simulated 600 target trajectories with launch speeds ranging from 0.1 to 60°/s in 0.1°/s steps. We then determined the trajectory (Fig. 1*A*, red dashed line) that produced the smallest Euclidian distance to the interception position. The corresponding target speed that best fit the observed interception position was labeled the estimated speed for that trial. This analysis assumes that observers correctly associate different launch speeds with the different target trajectories (Fig. 1*C*). We confirmed this assumption by analyzing both the vertical and horizontal interception errors, which directly reflect extrapolation errors (de la Malla et al., 2018).

The same analysis was repeated using the eye position at the time of interception to compare how well target speed was estimated based on hand and eye interception. In trials in which observers made a saccade at the time of interception, we used eye position at the offset time for this saccade for the analysis. Next, we analyzed saccade amplitudes during each trial to later obtain a readout of predicted target trajectories at different time points (see below). On average, observers made 2.8 ± 0.7 (mean ± SD) saccades during a trial. We analyzed the amplitude of the first catch-up saccade after target onset as an indicator of early trajectory estimation. After the first catch-up saccade, and after the target disappeared, observers typically made one or two subsequent saccades that brought the eye to the predicted interception location. To account for the varying number of saccades during this later phase of the trial, we calculated the cumulative saccade amplitude (i.e., sum of amplitudes of all subsequent saccades in a trial) as an indicator of late trajectory estimation.

### Statistical analyses

To assess effects of sound volume and visual presentation duration on our dependent variables—speed estimates based on interception end points and vertical saccade amplitudes—we first applied a within-subject *z* score outlier detection (data points were excluded if they were >3 SDs from an observer’s mean). We then calculated observers’ means per condition and fed the data into a repeated measures (rm) ANOVA with an alpha level of 0.05. To correct for multiple comparisons within multiway ANOVA, we applied a sequential Bonferroni correction (Cramer et al., 2016); a Bonferroni correction was also applied to all *post hoc* comparisons (two-sided, paired *t* tests).

In addition to testing these main effects, we also assessed whether physical target speed predicted estimated speed by applying a linear mixed model with physical target speed (continuous predictor), visual presentation duration (categorial predictor), and the interaction term as both fixed and random effects and observers as grouping variable. A linear mixed model was used to obtain regression slopes between physical target speed and speed estimates and to test whether speed estimates scaled more accurately with physical target speed when targets were presented for 300 ms versus 100 ms. All statistical analyses were performed in R software (R Core Team, 2022; www.r-project.org) using RStudio (http://www.rstudio.com/) and the afex (https://CRAN.R-project.org/package=afex), dplyr (https://CRAN.R-project.org/package=dplyr), and ez (http://github.com/mike-lawrence/ez) packages.

## Results

Observers tracked the brief launch of the simulated baseball and then intercepted it with a quick pointing movement along the predicted trajectory within a hit zone (Fig. 2*A*). In our task, observers had to rely on visual information of target speed during the brief visual presentation of the ball to extrapolate and intercept it accurately. We predicted that the brief visual presentation durations of 100 or 300 ms would result in conditions of low (short presentation) and high (longer presentation) visual certainty.

Mean 2D interception positions show that observers intercepted targets along their predicted trajectories and discriminated between different target trajectories in both the 100 ms (Fig. 2*B*) and 300 ms conditions (Fig. 2*C*). However, interception end points strongly regressed toward the intermediate trajectory in the 100 ms condition, indicating that observers were uncertain about the target trajectory. In contrast, in the 300 ms condition, observers intercepted balls more accurately along their trajectories (Fig. 2*C*).

### Auditory cues bias target speed estimates when visual information is uncertain

**Figure 3.**
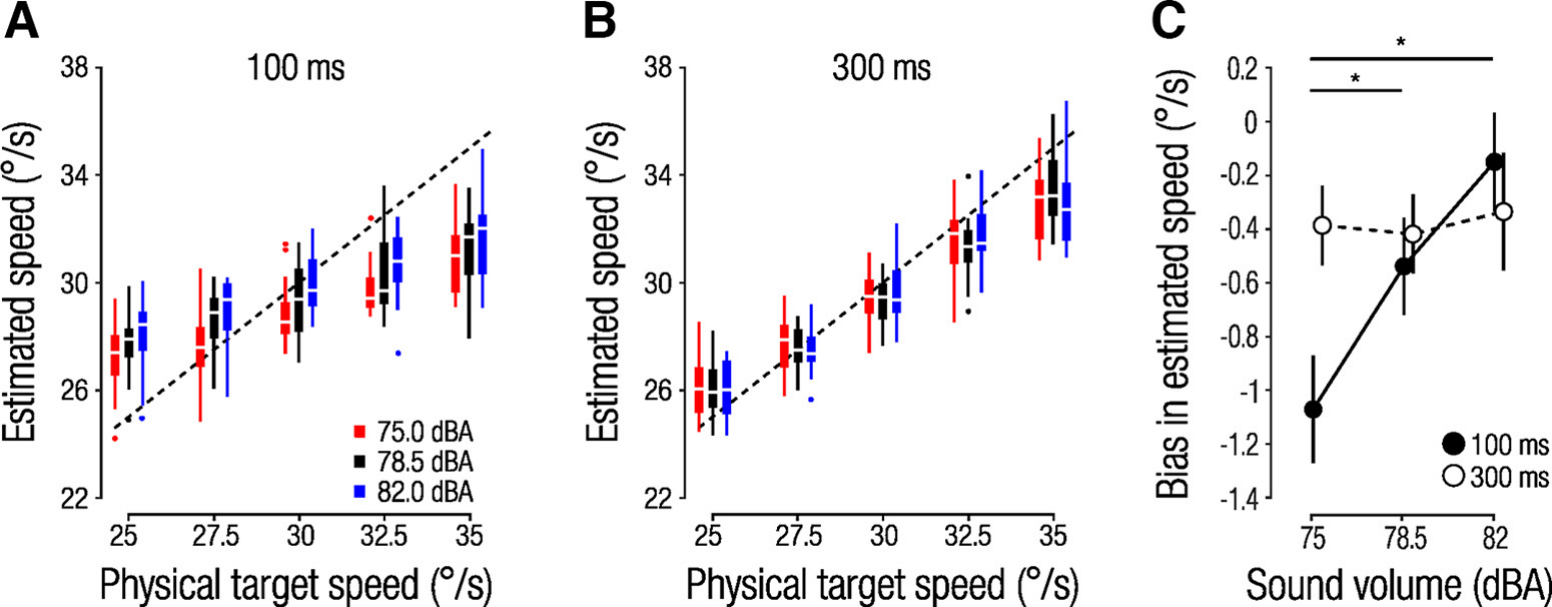
***A***, ***B***, Box plots of estimated target speed (*n =* 16) as a function of physical target speed. Colors denote sound volume conditions, and dashed lines indicate veridical estimates. ***A***, 100 ms condition; ***B***, 300 ms condition. ***C***, Effect of sound volume on the bias in estimated speed averaged across physical target speeds, separately for the 100 ms (filled circles) and 300 ms (open circles) condition. Circles and error bars denote the mean ± 1 within-subject standard error of the mean (SEM); significant *post hoc* comparisons, *p < 0.05).

We predicted that sound volume of the bat-ball contact would systematically bias observers’ speed estimates (quiet sounds indicating a softer hit and lower launch speed; loud sounds indicating harder hits and higher speed). Perceptual studies on multisensory cue combination indicate that sensory cues are weighed according to their uncertainty. We thus predicted that under high visual uncertainty (short visual presentation duration), target speed estimates show a systematic sound-induced bias. Under high visual uncertainty, observers are known to rely more strongly on the average speed of all physical targets when judging their trajectories (Jazayeri and Shadlen, 2010; Petzschner et al., 2015). We would therefore expect poor scaling of speed estimates with physical target speed (i.e., a strong center bias) in addition to the systematic sound-induced bias. Conversely, under low visual uncertainty, we expect speed estimates to scale more accurately with physical target speed (i.e., weak center bias) and to be less influenced by the auditory cue (Fig. 1*B*). To test these predictions, we measured observers’ speed estimate as the primary outcome measure. Figure 3 shows observers’ estimated speed as a function of physical target speed, separately for each sound volume. If speed estimates were accurate, they would fall along the diagonal (dashed line). First, we ran a linear mixed model with physical target speed as a continuous predictor and visual presentation duration as a categorial predictor. Physical target speed was a significant predictor of estimated speed for both visual presentation durations (100 ms, *β* = 0.37, *t*_(15)_ = 9.66, *p <* 0.001; 300 ms, *β* = 0.72, *t*_(15)_ = 14.94, *p <* 0.001). In line with our predictions, we found a significant difference between slopes for the 100 and 300 ms conditions (*β* = −0.35, *t*_(15)_ = 15.92, *p <* 0.001), confirming that observers’ speed estimates regressed more toward the mean (indicating high visual uncertainty) in the 100 ms condition compared with the 300 ms condition. Accordingly, the mean 2D interception error was higher in the 100 ms (2.66° ± 0.46°) compared with the 300 ms condition (2.06° ± 0.44°; *t*_(15)_ = 10.86, *p <* 0.001). Together, these findings show that an additional 200 ms of target visibility provide significantly more visual information used to enhance observers’ speed estimates.

Next, we asked whether and under which conditions sound volume influenced speed estimates. We hypothesized that sound volume would systematically bias observers’ speed estimates and that this bias would depend on the certainty of the visual speed signal. Accordingly, we found that observers systematically underestimated speed when the ball launch was paired with a quiet batting sound and overestimated speed when the ball was paired with a loud batting sound. This effect was consistent across all target speeds at short visual presentation duration (Fig. 3*A*). Conversely, at long visual presentation durations, sound volume did not systematically affect estimated speed (Fig. 3*B*). To assess the differential effects of sound volume at different visual presentation durations we calculated each observer’s bias in speed estimation across physical target speeds (mean difference between estimated speed and physical target speed; Fig. 3*C*). A 2 (visual presentation duration) × 3 (sound volume) rmANOVA revealed a significant main effect of sound volume (*F*_(2,30)_ = 4.91, *p =* 0.029, *η_p_*^2^ = 0.25) and no main effect of visual presentation duration (*F*_(1,15)_ = 0.60, *p =* 0.45, *η_p_*^2^ = 0.04). A significant sound volume × visual presentation duration interaction (*F*_(1.43,21.46)_ = 20.30, *p <* 0.001, *η_p_*^2^ = 0.58) confirmed the profound effect of auditory cues on manual interception when visual information is sparse but not when the target is presented sufficiently long to base speed estimation for interception on visual information alone. These findings show that when visual information was sparse and thus uncertain, speed estimates were strongly biased toward the mean and systematically influenced by the auditory cue. Conversely, when visual uncertainty was low, estimated speed scaled almost perfectly with physical target speed (weak center bias) and showed no impact of auditory cues.

### Eye movements reveal temporal dynamics of audiovisual integration

The extent to which observers relied on sound depended on the certainty of the visual speed signal, that is, visual presentation duration (low certainty for short, high certainty for longer presentations). The impact of the auditory signal decreased with increasing visual presentation duration. To assess how differences in auditory signal use in the long and short visual presentation duration conditions unfolded over time, we analyzed observers’ continuous eye movements during the interception task.

**Figure 4.**
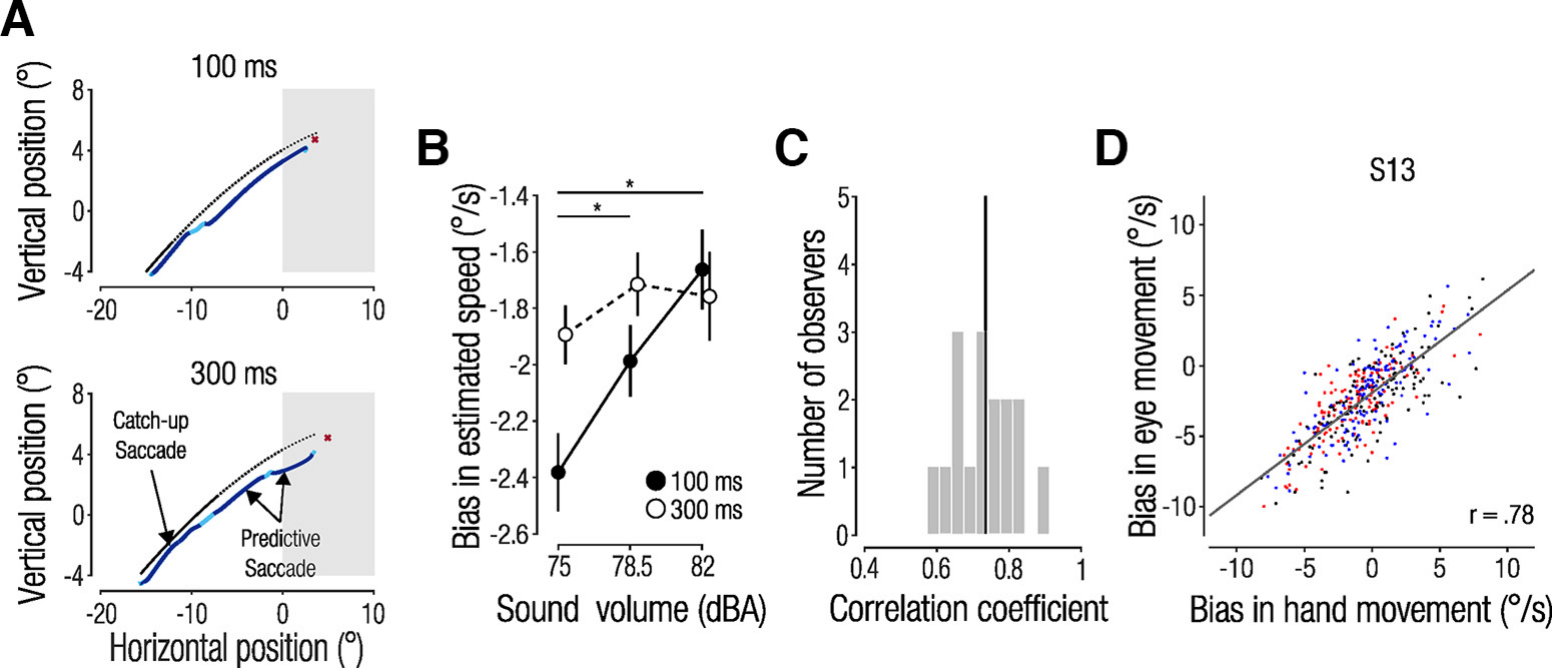
***A***, Two-dimensional eye position traces of two representative trials. Bright blue segments indicate smooth pursuit, continuous tracking of moving targets with the eyes, and dark blue segments indicate saccades. Solid and dashed black lines represent the visible and invisible portions of the target trajectory. The shaded area represents the hit zone. ***B***, Effect of sound volume on estimated speed based on final eye position. ***C***, Histogram of trial-by-trial correlation coefficients from all observers. Black line indicates mean across observers. ***D***, Trial-by-trial correlation of one representative observer.

Observers tracked the simulated baseball with their eyes using a combination of smooth pursuit and saccadic eye movements (Fig. 4*A*). They typically made an early catch-up saccade shortly after target onset (mean = 125, SD *=* 38 ms). Subsequent predictive saccades were made after target disappearance to the predicted interception location. Eye movement endpoints, based on the 2D eye position at the time of interception, reflect observers’ speed estimates. Figure 4*B* shows that observers underestimate speed in the presence of a quiet sound and overestimate speed when paired with a louder sound, akin to observations for manual interception responses (Fig. 3*C*). Accordingly, speed estimates based on eye and hand movement end points were strongly correlated on a trial-by-trial basis with a mean correlation of *r =* 0.73 (measured across physical target speeds and sound volumes; Fig. 4*C*; trial-by-trial correlation of one representative observer depicted in Fig. 4*D*).

We next assessed whether eye movements can indicate the time point at which the auditory cue first started influencing observers’ trajectory estimates. Specifically, we asked whether the first catch-up saccade made after target onset (initiated with a mean latency of 125 ms) was already influenced by sound volume. This would indicate early audiovisual integration. By contrast, an effect only on subsequent predictive saccades, made later in the trial, would indicate that integration processes take longer. We analyzed the amplitude of the first catch-up saccade and the combined amplitudes of subsequent, predictive saccades. If sound volume biases saccades similarly to what we observed for eye and hand interception end points, we would expect that loud sounds lead to larger saccades (following a trajectory with higher launch speed), and that quiet sounds lead to smaller saccade amplitudes. For these analyses, we excluded trials where the first catch-up saccade was made in anticipation of target onset (≤50 ms latency, 3.9% of trials).

**Figure 5.**
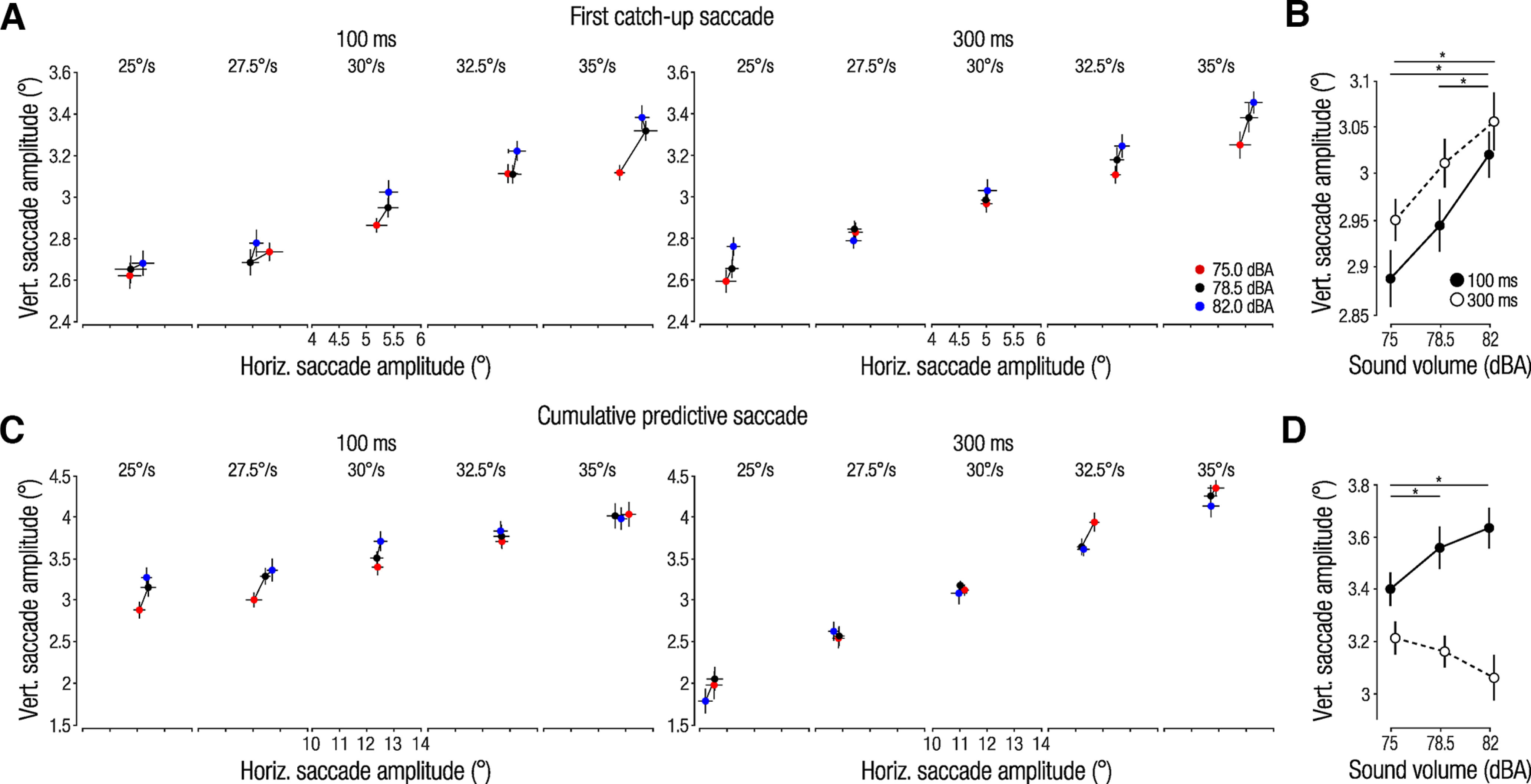
Saccade analyses. ***A***, Effect of sound volume on horizontal and vertical saccade amplitudes for the first catch-up saccade after target onset. ***B***, Vertical saccade amplitudes averaged across physical target speeds. ***C***, ***D***, Cumulative saccade amplitudes of all subsequent saccades. Circles and error bars show means ± 1 within-subject SEM; significant *post hoc* comparisons, *p < 0.05. Note different scales between top and bottom panels.

Figure 5*A* shows the horizontal amplitude of the first catch-up saccade plotted against the vertical amplitude, separately for each physical target speed and for the two visual presentation durations. We found an influence of sound volume on the amplitude of the first catch-up saccade, consistently observed across physical target speeds and visual presentation durations. Sound volume exhibited the strongest influence on the vertical saccade amplitude, in line with our observation that interception end points differentiated between trajectories primarily along the vertical axis (Fig. 2*B*,*C*). Feeding the mean vertical saccade amplitudes (averaged across physical target speeds) into a 2 (visual presentation duration) × 3 (sound volume) rmANOVA revealed a main effect of sound volume (*F*_(2,30)_ = 26.24, *p <* 0.001, *η_p_*^2^ = 0.64; Fig. 5*B*). Neither the main effect of visual presentation duration nor the interaction term were significant (all *p *values >* *0.388), indicating that the auditory cue influenced speed estimates early during the trial and before any differences in presentation duration could have had an impact on these estimates. Note that the first catch-up saccade not only showed consistent and similar effects of sound volume between presentation durations but also scaled similarly with physical target speed (Fig. 5*A*). This further indicates that early catch-up saccades were finely tuned to the sensory properties of the target and were programmed before differences between presentation durations emerged.

This early auditory bias, observed for both visual presentation durations, contrasts with our finding that speed estimates based on eye and hand movement end points were only biased for the short but not the long duration. We would therefore expect that subsequent, predictive saccades reverse the early auditory influence when more visual information is available (i.e., in the 300 ms condition). Thus, we next analyzed the combined amplitudes of all subsequent saccades. We used the cumulative saccade amplitude because the number of saccades differed between trials and observers, meaning that a reversal of the early auditory influence could either occur by making smaller or fewer saccades. In line with our expectation, predictive saccades that occurred later in the trial had larger amplitudes with increasing sound volume in the 100 ms condition but smaller amplitudes with increasing sound volume in the 300 ms condition (Fig. 5*C*). Again, sound primarily affected the vertical component of the cumulative saccade amplitudes.

We averaged vertical cumulative saccade amplitudes across physical target speeds (Fig. 5*D*) and fed the data into a 2 (visual presentation duration) × 3 (sound volume) rmANOVA. In line with our expectation of a differential impact of sound volume, depending on availability of visual information, we did not find a main effect of sound volume (*F*_(1.38,20.68)_ = 0.71, *p =* 0.454, *η_p_*^2^ = 0.04) but instead a strong visual presentation duration × sound volume interaction (*F*_(2,30)_ = 20.46, *p <* 0.001, *η_p_*^2^ = 0.58). A significant main effect of visual presentation duration (*F*_(1,15)_ = 10.98, *p =* 0.009, *η_p_*^2^ = 0.42) is likely because of smaller saccades in the 300 ms condition, which generally elicits stronger pursuit. The differential impact of sound volume for the 100 and 300 ms conditions indicates a reversal of the early auditory influence with the availability of additional visual information. This observation was further supported by the finding that predictive saccades in the 300 ms condition scaled more with physical target speed than predictive saccades in the 100 ms condition (Fig. 5*C*).

## Discussion

**Figure 6.**
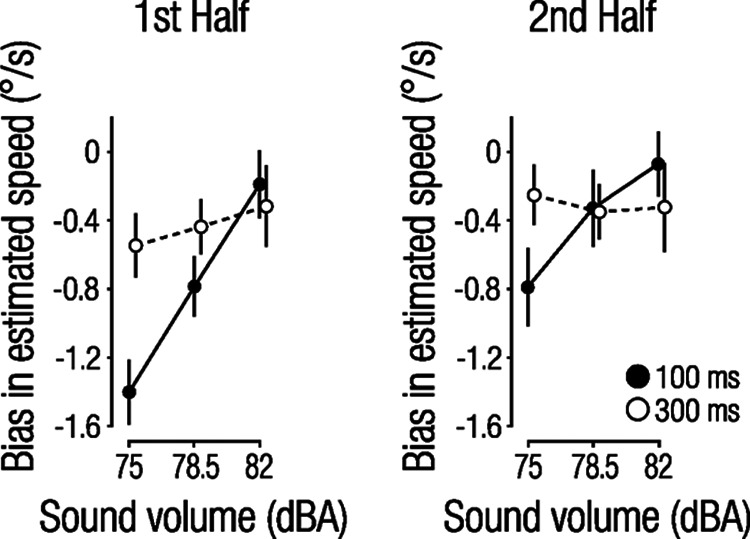
Bias in estimated speed split separately for the first and second half of the experiment. Solid lines and filled circles represent 100 ms, and dashed lines and open circles represent the 300 ms condition. Circles and error bars show mean ± 1 within-subject SEM.

Predicting the trajectory of a moving object is a fundamental ability that allows us to accurately hit, catch, or otherwise intercept targets (Fiehler et al., 2019). Most research on interception focuses solely on vision to form trajectory predictions and guide interceptive hand movements (Brenner and Smeets, 2018; Fooken et al., 2021). Yet, in our natural environment, object motion is typically accompanied by sounds that can provide additional information about the motion of an object. Here, we show that auditory signals are used in combination with visual motion information to estimate target speed for interceptive actions. Using a rapid track-intercept task in which a visual trajectory was paired with batting sounds of varying intensities we present three key findings. (1) Sound volume of bat-ball contact systematically influences interception responses, extending well-known effects of audiovisual integration on perception to interceptive actions. (2) Integration of auditory cues and visual information depends on the certainty of the visual signal; auditory cues influence speed estimates only when visual information is sparse. (3) Audiovisual integration occurred as early as the first catch-up saccade (initiated 125 ms after target onset on average); with the availability of additional visual information, the early sound bias was reversed. The temporal dynamics of audiovisual integration was revealed by analyzing continuous eye movements during this task. In our experiment, sound volume was never informative of physical target speed, precluding the possibility that our results were solely caused by learning to associate certain sound volumes to certain target trajectories. Instead, our findings likely reflect a natural association between sound volume and relative target speed gained through lifelong experience. Under similar environmental conditions, particularly when the target is always at the same distance from the observer, louder sounds will typically correspond to higher target speeds. When splitting our data between first and second halves of the experiment, we found that the auditory influence was stronger during the first half of the experiment (Fig. 6). This indicates a strong association between sound volume and target speed that decreased with increasing task experience. Together, these findings highlight the important contribution of auditory cues for vision-guided actions, particularly in situations where visual information is sparse or uncertain. These results build on a long line of literature on audiovisual signal integration for perceptual tasks (Ernst and Bülthoff, 2004). The novelty of our findings lies in uncovering how auditory information contributes to vision-guided interception, a fundamental ability for everyday interactions.

By manipulating the visual presentation duration of the target, we revealed that the use of auditory cues critically depends on the uncertainty of the visual motion signal. This finding is aligned with previous perceptual studies on multisensory cue combination that used Bayesian observer models and show that prior information and sensory evidence are combined depending on their respective uncertainty (Ernst and Banks, 2002; Alais and Burr, 2004; Körding et al., 2007; Angelaki et al., 2009). Congruently, we found that speed estimates were only influenced by auditory cues when visual information was sparse, whereas the auditory cue was largely ignored when sufficient visual information was provided. Moreover, we observed a strong center bias in speed estimates when visual information was uncertain. This type of finding is typically interpreted to indicate use of a prior based on the statistics of the stimuli used (Jazayeri and Shadlen, 2010; Petzschner et al., 2015; Chang and Jazayeri, 2018). Alternatively, priors can also be derived from statistics of our natural environment. Studies on visual (Stocker and Simoncelli, 2006) and auditory motion perception (Senna et al., 2015) revealed that observers typically rely on a slow-motion prior. Our finding that observers generally undershot target trajectories (Fig. 3) fits with those results.

It is important to note that any variation in ball presentation duration might not only affect visual uncertainty but might also impact the reliability of the auditory cue. The auditory cue was always presented at the time of ball launch, whereas visual information was either presented for 100 or 300 ms. Therefore, a longer visual presentation might potentially downweigh the reliability of the auditory cue as more visual information was provided after the sound. Because we did not independently manipulate the reliability of both cues, we cannot rule out that the reliability of the auditory cue might have had an impact on our results.

Whereas our approach did not allow us to fully test Bayesian cue integration, future studies could include unimodal (auditory and visual) conditions in addition to the audiovisual condition to directly test predictions of Bayesian cue combination in the context of interception. Moreover, including an auditory-only condition could allow assessment of whether observers naturally associate auditory intensities of bat-ball contact with ball launch speed even in the absence of visual information.

Our interception task was inspired by baseball. We used a visual target that moved along a simulated batted baseball and a naturalistic batting sound. Based on real-world Major League Baseball data, it was recently shown that baseball batters rely on prior knowledge and visual cues, for example, a pitcher’s posture and hand position when estimating where to swing (Brantley and Körding, 2022). Simple cues and heuristics are critical in baseball, where hitters only have a few hundred milliseconds to decide whether and where to swing (Gray and Cañal-Bruland, 2018). In this or similar rapid decision-making contexts, auditory cues may provide a critical advantage because combining them with visual cues can reduce uncertainty (Alais and Burr, 2004). Yet, future studies are needed to assess whether athletes rely on auditory cues of bat-ball contacts, in addition to prior knowledge and visual signals during real-world interceptive sports, as our findings suggest.

### Eye movements as sensitive indicators of audiovisual integration

Eye movements are a natural, instinctive behavior in tasks that require fine-motor interactions with a visual object. When manually intercepting, hitting, or catching an object, observers track its trajectory until the point of interception (Mrotek and Soechting, 2007; Fooken et al., 2021). The continuous nature of these movements provides an opportunity to relate their kinematics to ongoing cognitive task processes, such as decision-making (Spering, 2022). Here, we used observers’ continuous eye movements to probe the temporal dynamics of audiovisual integration. We observed a systematic influence of the auditory cue on the first catch-up saccade, which was initiated, on average, 125 ms after target onset. At this early time point, louder sound volumes evoked larger saccade amplitudes. If additional visual information was available (long visual presentation duration), subsequent saccades reversed this early auditory effect. This finding suggests that the integration of auditory and visual signals can occur at a very short timescale, in line with findings showing early effects of audiovisual cues on pupil dilation and simple saccadic decision-making (Wang et al., 2017). Previous studies have identified the superior colliculus—a midbrain structure that is also involved in the control of eye movements (Sparks, 1999)—as a key hub of audiovisual integration (Stein and Stanford, 2008). Visual and auditory signals reach this brain structure within 80 ms (Ito et al., 2021), making this area an excellent candidate for short-latency audiovisual integration. In parallel, visual and auditory signals could also be integrated in cortical sensory areas such as the middle temporal cortex, an area traditionally dedicated to early visual motion processing (Rezk et al., 2020).

We conclude that auditory signals significantly and systematically have an impact on vision-guided interceptive actions. This influence was strongest when visual information was sparse. We show that noninvasive, time-sensitive eye movement measurements can provide new behavioral evidence for early and rapid integration of auditory and visual signals.

## Synthesis

Reviewing Editor: David Franklin, Technische Universitat Munchen Decisions are customarily a result of the Reviewing Editor and the peer reviewers coming together and discussing their recommendations until a consensus is reached. When revisions are invited, a fact-based synthesis statement explaining their decision and outlining what is needed to prepare a revision will be listed below. The following reviewer(s) agreed to reveal their identity: Jeroen Smeets. Note: If this manuscript was transferred from JNeurosci and a decision was made to accept the manuscript without peer review, a brief statement to this effect will instead be what is listed below.

Your manuscript has been reviewed by two experts in the field. They both agree that the study of the combination of auditory and visual signals during manual interception tasks is of interest. However, we all have concerns with the current manuscript. Specifically, there are major concerns with the claim of Bayesian integration when you do not measure the independent effects of auditory and visual signals, and the temporal effects of the signals on the integration of the information. In addition, there are many issues that both reviewers point out regarding the current manuscript, which are detailed in the independent reports below. I encourage you to revise your manuscript, taking into account each of the concerns raised by the reviewers, and preparing a point-point-response.

Reviewer 1

Summary

The authors here examine whether auditory signals are considered when intercepting a visual target. Participants intercepted at a designated area a virtual disk that was moving at different speeds. The disk was visible only for the first 100 or 300 ms after its launch. Simultaneously with the disk’s launch, a batting sound of various intensities was heard, but importantly this sound was never informative about the disk’s speed.

The key finding of this study is that interception was affected by the auditory signal. As it appears, participants intercepted the disk at an earlier spatial location when the disk’s launch was paired with a louder sound, indicating that a louder sound biases participants to believe that the disk moves faster. This effect was evident only when the disk was visible for a shorter (100 ms) than a longer (300 ms) duration after its launch. The authors conclude that this selective influence of the additional auditory cue on interception is based on a Bayesian integration of the available sensory signals, in the sense that when the visual input is less reliable (i.e. 100 ms presentation duration), then the auditory cues are upweighted and influence related motor behavior.

Main evaluation

The study is novel in the sense of examining the role of redundant sensory signals on interceptive behavior. The background is presented in a fair way and the methods are described sufficiently to reproduce the study, although some methodological decisions confused me. The results are generally easy to follow but some parts can be presented more clearly and some additional information can assist in comprehension. Some of the conclusions need to be more carefully expressed -for instance, the current study design does not allow to fully claim that there is Bayesian integration in the tested paradigm.

Specific comments

The authors claim that the findings resemble a Bayesian integration of auditory and visual signals related to disk interception. I acknowledge that the authors are at places careful in how they formulate this statement, however the conclusions can get misleading because the current design does not allow to fully test a possible Bayesian integration. The reason is that, for that purpose, two unimodal conditions would be necessary: a pure auditory condition and a pure visual condition, through which one could calculate the reliability of the related sensory cue.

The manipulation to induce visual uncertainty is also somewhat confusing and I would like to kindly ask the authors for some elaboration on the idea. I understand that with longer presentation times of the moving disk, one can obtain richer visual feedback about the disk’s trajectory. However, I was expecting a manipulation that would keep the duration of presentation identical while inducing variability at the instantaneous properties of the disk. With the current design, one condition provides 200 ms longer visual input than the other. This raises a number of issues that need clarification.

(1) In the longer presentation time (300 ms), the most recent visual input occurs ∼200 ms after the auditory cue. This temporal deviation of 200 ms (compared to the 100 ms condition) could explain the absence of audiovisual integration: the auditory cue in the 300 ms condition occurred much earlier in the past and therefore the conveyed information decayed over time, becoming less trustworthy, always relative to the 100 ms condition. Although this would still be in line with a Bayesian integration account, the emphasis here would be in the reliability of the auditory cue, and not of the visual cue.

(2) Richer visual feedback from the longer target presentation does not necessarily mean more informative visual feedback. Is there evidence that this 200 ms can be more informative, which can justify the decision for this manipulation? For instance, Lopez-Moliner and colleagues (2010, EBR) suggest that the most informative aspect when intercepting a moving target is the time when the target starts moving, and in this experiment both visual conditions cover this period. I acknowledge the methodological differences between that and the current study, but the important aspect is on the contribution of this additional 200 ms.

(3) What information is provided by the visual feedback? The authors mention that longer visual feedback provides better speed estimates of the disk. Is therefore speed the critical factor to intercept the moving object? For instance, goal-directed hand trajectories are based on the target’s positional information, whereas target speed influences the velocity profile of these trajectories (Smeets & Brenner, 1995, JEP:HPP). Would the authors expect similar results on audiovisual integration should they had induced uncertainty by illusory changes in speed, say through background motion?

I suggest that the authors report (a) whether participants were more successful in hitting the target in the 300 ms than in the 100 ms condition, and (b) the interception moment relative to disk movement onset for each visual condition. The former could give insights as to whether the additional 200 ms were useful for the interception task. The latter could indicate whether motor output (interception) is related to when the sensory input was provided. In other words, did participants intercept earlier in the 100 ms condition to reduce the time during which no auditory/visual cue is available?

It is unclear why the authors use ANOVAs for some tests and LMMs for some other tests. This should be explained in a comprehensive way. For instance, LMMs could model trial-by-trial variability that cannot be considered when feeding averages in ANOVAs. I am not sure if this is what the authors did or aimed for, so it should be explained.

Line 281ff: This section has confused me. First, the authors need to make more explicit how exactly eye movements can reveal the time course of a possible audiovisual integration. Second, it is unclear why the authors consider only the vertical amplitude of catch-up and predictive saccades for the related analyses (lines 282-284)? The disk is moving along a parabolic trajectory, so both its vertical and lateral position are relevant. Third, why is the “early timepoint” (line 283) chosen at 125 ms after disk movement onset? Related to this, the authors write that this early time point refers to saccades “initiated ∼125 ms after target onset”. What does this approximation mean practically?

Minor comments

Lines 266-277: What did the authors determine the eye movement endpoint if the eyes were moving at the moment of interception?

Figure 4: I guess that the grey area is the interception area. Could the authors please clarify this in the figure’s caption?

Line 339: This is true under the assumption that the disk is at the same distance from the observer. It should not affect the current data, but this sentence may require this small clarification.

Line 368-369: What does the activity in the hippocampus tell us? Why is this sentence relevant here?

Reviewer 2

I (Jeroen Smeets) sign this review, as I will cite my own work to make my arguments. This is in no way intended as a request for citations by the authors.

The authors address an interesting question, whether one integrates visual and auditory information when intercepting a moving target. The authors convincingly show that the intensity of a sound at the moment of movement onset of the target biases the predictions of the target’s motion, especially if the information of the target’s motion is poor. However, in some places, the authors over-interpret their findings.

This is quite a complicated question, as this involves three signals: position, time, and motion. Most importantly: these three sources of information are related physically, but not necessarily perceptually (Smeets & Brenner, 2023). For intercepting a visual target, for instance, we showed very recently that when intercepting a moving target, people update motion information in a different way than position information (Brenner, de la Malla, & Smeets, 2023). Most importantly, people use information from previous trials to compensate for their inability to use visual information on acceleration (Brenner et al., 2016). The analysis of the authors assumes that participants only have errors in their judgement of velocity, not in position or acceleration. Auditory motion perception is much less studied than visual motion perception, but there are several studies more relevant than some of the references cited in l42-43. The authors themselves cite in the discussion for instance Senna et al (2015); a useful review might be the one by Carlile and Leung (2016).

I find the Bayesian integration approach of the authors a bit problematic. To be able to use Bayesian integration of two modalities, one needs to be able to express the two signals themselves in the same space. The authors do so in their figure 1B, but it is unclear to me what the evidence is that the sound volume provides a percept of speed. Why didn’t the authors use an actual simulation of motion? Moreover, I do see no attempts to determine the bias and precision of the visual and auditory motion, so a proper Bayesian analysis is impossible. A second limitation of the Bayesian analysis by the authors is that they neglect that it does not only predicts a change in the bias, but also a change in precision. Unfortunately, the authors did not use a condition without sound (and, as mentioned above, a condition without vision). In the discussion, the authors even state (L334): “In our experiment, sound intensity was never informative of physical target speed”, so apparently they agree with my observation that Bayesian integration is not applicable here. I would therefore suggest leaving out any claims on Bayesian integration, and just referring to influence as a ‘multisensory interaction’.

The authors do not present the most straightforward analysis of interception: the horizontal error between the interception point and the position of the target at the moment of interception. This measure directly reflects the horizontal component of the extrapolation error (de la Malla, Smeets, & Brenner, 2018), and thus the bias in motion perception. The advantage of this measure above the one the authors use is that it does not involve an assumption about acceleration (only position and time). If this analysis yields another value for the bias in speed than that of the authors, some of the assumptions underlying their analysis might be incorrect. For the eye-movement data, an analysis of the horizontal component would be useful as well. It is not clear to me why they analyse the data of the eye in a different way than the data of the hand. I personally find Figure 5 the most convincing of the paper. Adding the horizontal component to this figure and using the same format for the manual interception position might be informative. Instead of the first saccade, one could take the hand position at some earlier moment.

A last general aspect is the time course of the integration. It is not clear to me what the authors mean by this concept, as both the visual and auditory information are available for only a very brief moment. Especially for the 100 ms presentation duration, there is not much room for a time course. The authors argue that the 100 ms presentation duration implies uncertainty. Indeed, as this duration is likely to be close to the minimal delay of motion detectors (at least for some participants; van de Grind, Koenderink, & van Doorn, 1986), so the uncertainty is maximal.

Details:

L9 I initially misunderstood “presentation time”: not as the duration of the presentation, but the moment of presentation (e.g. starting at motion onset, or starting somewhat later). Probably use “presentation duration”?

L44 “Volume” is an ambiguous concept. Please explain somewhere what you use: absolute sound pressure levels, or perceptually normalized ones.

L53: unclear why you cite here Metzger (1934) as a reference for ambiguity due to visual illusions. This paper discusses the same ambiguous stimulus as discussed by Sekuler et al (and is the first reference in that paper), but ambiguous event perception (balls colliding or passing) has no relation with the topic of optimal cue combination or Bayesian integration as it is used in the present paper.

L110: The paper does not mention that the balls are simulated as spinning, so how can there be a Magnus effect?

L126 “occlusion” was not mentioned. Do you refer to the “disappearing” in line 121? Try to be consistent.

L129 “Condition” not defined. Please make explicit what the 30 conditions are. Also, explain what happened between the blocks.

L136: “saccade on- and offsets were then determined as the nearest reversal in the sign of acceleration” This sounds incorrect, as the method starts by finding a moment where the velocity surpasses some threshold. From that moment, one can go back in time, and the first reversal might be regarded as the moment of saccade onset. But when moving in the other direction, the nearest reversal is the moment of peak velocity, which is a rather strange definition of saccade offset.

L138: strange to up-sample from 120 to 1000 Hz before low-pass filtering at 15 Hz. This will create artefacts.

L151 Please specify the launch angle (I assume that is constant).

L153 (and L208) “(dashed red line in Fig.1A)” seem to refer to the “shortest Euclidian distance”, but it refers to “trajectory”. Better reposition this phrase. By the way: I don’t understand why the authors use a simulation: it is possible to directly calculate the speed that brings the ball to a specific position.

L163 the wording “effects of stimulus volume and presentation time” does not convey that the former aspect is auditory (i.e. not ball volume) and the latter aspect is only visual (the auditory presentation time was not varied). Better choose a more intuitive naming of the dependent variables.

L168 Confusing to alternatingly use “target speed” and “physical target speed”

L190, figure 2B: It is unfortunate that you cannot see which data points belong to the same participant. It seems as if the within-participant structure of the hitting positions (higher speed

Figure 4A: please indicate which part of the trajectory was visible.

L264: please use fewer decimals, using “124{plus minus}38 ms” contains all relevant information.

L271 “correlated on a trial-by-trial basis with a median” I guess this is one value across all conditions (target velocities and sound levels)? Please make this explicit.

L272 “with a median” Unclear why here a median is presented, and all other measures are presented as means. Probably, a scatterplot for a representative subject (one with r=0.7) would be informative (reducing the size of the histogram), especially if the sound-level would be indicated using the red-blue-black coding as in figure 3AB.

L283 “∼125 ms” This number occurs also in the discussion and even makes it to the abstract, but the authors do not explain how this value was determined.

L312 “cumulative” would be nice if this measure (and actually: the whole analysis of the eye-data) would have been explained in the methods.

L332 “is corrected” I don’t think I have seen any evidence for ‘correction’, the authors only report evidence for accumulation of speed information over time, in line with our results (Brenner et al., 2023).

References

Brenner, E., de la Malla, C., & Smeets, J. B. J. (2023). Tapping on a target: Dealing with uncertainty about its position and motion. Experimental Brain Research, 241(1), 81-104. doi:10.1007/s00221-022-06503-7

Brenner, E., Rodriguez, I. A., Munoz, V. E., Schootemeijer, S., Mahieu, Y., Veerkamp, K., . . . Smeets, J. B. J. (2016). How can people be so good at intercepting accelerating objects if they are so poor at visually judging acceleration? I-Perception, 7(1), 1-13. doi:10.1177/2041669515624317

Carlile, S., & Leung, J. (2016). The perception of auditory motion. Trends in Hearing, 20, 19. doi:10.1177/2331216516614254

de la Malla, C., Smeets, J. B. J., & Brenner, E. (2018). Errors in interception can be predicted from errors in perception. Cortex, 98, 49-59. doi:10.1016/j.cortex.2017.03.006

Smeets, J. B. J., & Brenner, E. (2023). The cost of aiming for the best answers: Inconsistent perception. Frontiers in Integrative Neuroscience, 17, 13. doi:10.3389/fnint.2023.1118240

van de Grind, W. A., Koenderink, J. J., & van Doorn, A. J. (1986). The distribution of human motion detector properties in the monocular visual field. Vision Research, 26(5), 797-810. doi:10.1016/0042-6989(86)90095-7
